# Acute effects of Nitrosigine® and citrulline malate on vasodilation in young adults

**DOI:** 10.1186/s12970-020-00343-y

**Published:** 2020-02-24

**Authors:** Jeffrey M. Rogers, Joshua Gills, Michelle Gray

**Affiliations:** Exercise Science Research Center, University of Arkansas, 1 University of Arkansas, HPER 321-E, Fayetteville, AR 72701 USA

**Keywords:** Citrulline malate, Arginine silicate, Nitrosigine®, Flow mediated dilation, Vasodilation

## Abstract

**Background:**

Athletes are increasingly exploring ways to enhance their physical performance. Increasing blood flow to the working tissues through endothelium-dependent vasodilation is one factor athletes use to realize these results. Sports supplements such as pre-workouts tout this benefit; however, many have not been tested under laboratory conditions to examine the effects of commonly used supplements on vasodilation. Two popular supplements are Nitrosigine® and citrulline malate (CM). Thus, the purpose of this experiment was to determine the effects of Nitrosigine and CM on vasodilation using ultrasound and flow mediated dilation (FMD).

**Methods:**

Healthy, normotensive, and physically active male (*n* = 16) and female (*n* = 8) young adults participated in the present investigation. We utilized a randomized, double-blind, within-subjects design where participants reported for three trials, each preceded by a 7-day washout period. Baseline FMD measurement was obtained for each visit, followed by consumption of one clinical dose CM (8 g), Nitrosigine (1.5 g), or dextrose placebo (8 g). Following a 60-min digestion period, FMD was repeated. Supplementation order was randomized controlling for potential order effects.

**Results:**

Repeated measures ANOVA yielded a significant supplement (3) x time (2) effect (*p* < .001), such that Nitrosigine and CM yielded a greater improvement in FMD response than placebo. After supplementation, Nitrosigine and CM increased FMD by 31 and 34%, respectively, compared to a decrease of 2% during the placebo trial. After allometric scaling of the FMD values, supplement x time effect remained significant (*p* = .001) and changes were similar to non-scaled results. Nitrosigine (23%) and CM (25%) generated significantly greater allometric scaled FMD values when compared to the placebo trial (0.60%).

**Discussion:**

Both Nitrisigine and CM increased endothelial-dependent vasodilation as measured by a change in FMD. Increased vasodilation leads to an increase in skeletal muscle blood flow resulting in potential improvements in exercise performance.

## Background

The use of ergogenic dietary supplements has become increasingly popular among both recreational and competitive athletes, but the sale and use of such supplements has far outpaced evidence of their effectiveness. Substances that reportedly increase endothelium-dependent nitric oxide (NO) production are among the most commonly included in pre-workout supplement blends. By increasing NO production during exercise, athletes aim to experience greater muscle hyperemia and decreased muscular fatigue during resistance training. Until recent years, one of the most popular NO-boosting supplements was L-arginine, an amino acid used by the vascular endothelium in the synthesis of NO [1, 2]. Increased serum arginine concentration produces more NO; though, oral L-arginine supplementation is not an efficient mechanism of increasing serum arginine. After oral consumption, L-arginine must first be metabolized by the liver before entering circulation, rendering it less bioavailable and less effective than other substances at increasing NO production. Inositol-stabilized arginine silicate (Nitrosigine) and L-citrulline, have since replaced L-arginine in many pre-workout supplement blends. Comparison studies have found that supplementing with L-citrulline or Nitrosigine, more effectively elevates serum arginine and serum citrulline resulting in a greater potential increase in NO concentration [3–5]. Nutritional researchers have been particularly interested in L-citrulline because watermelon naturally contains dense quantities, but studies of watermelon juice and rind have determined that athletes would not be able to consume enough watermelon to experience significant ergogenic effects [6, 7].

Not only are Nitrosigine and L-citrulline more effective at increasing serum levels of arginine, but both could potentially provide ergogenic benefits beyond L-arginine supplementation [5, 8, 9]. Unlike dietary L-arginine, orally ingested L-citrulline bypasses liver metabolism after ingestion and is readily converted into L-arginine [2]. L-citrulline studies have found similar results to those administering intravenous L-arginine for increasing vasodilation leading many pre-workout supplements to now include a compound form that is commonly referred to as citrulline malate (CM). Recent research suggests that exercisers may experience added ergogenic benefits when compared to L-citrulline alone [10–14]. Comparable to supplementing with L-citrulline, clinical doses of CM produce the same level of NO during exercise [15]. Studies examining potential exercise benefits have found CM increases total work capacity during high intensity resistance exercise [11, 13, 14, 16], but ergogenic benefits are not only limited to anaerobic exercise. The malate component of CM may provide additional benefits to aerobic metabolism via an increase in the efficiency of oxidative adenosine triphosphate production, because of its intermediate role in the tricarboxylic acid cycle [10]. Significant ergogenic effects have been reported after acute and chronic supplementation of 3-8 g L-citrulline or CM [17]. Although CM enhances exercise performance, the effect on endothelium-dependent vasodilation has yet to be determined [18].

Though Nitrosigine was originally engineered as a dietary intervention for atherosclerosis and bone mineral density disorders, it can now be found in many pre-workout supplements as a vasodilator. Like L-citrulline, Nitrosigine supplementation increases exercise blood flow and decreases biomarkers of muscle fatigue following exercise [4]. Nitrosigine appears to lower systolic and diastolic blood pressure [19] and has gained a reputation in the pre-workout supplement market as a superior form of L-arginine because of the greater efficiency and length of time for which Nitrosigine increases serum arginine levels [20, 21]. Nitrosigine also out-performs L-arginine in terms of increased exercise blood flow and decreased biomarkers of muscle fatigue following exercise [4]. The known ergogenic effects of Nitrosigine are limited, but benefits similar to those found with CM supplementation (increased muscle hyperemia during exercise and decreased muscle damage biomarkers post-exercise) have been noted [4].

Although there is increasing evidence to support the presence of ergogenic benefits with CM and Nitrosigine supplementation, little research has examined their effects on endothelium-dependent vasodilation. Because there has been no investigation into the effects of CM and Nitrosigine on flow-mediated dilation (FMD) in active young adults, the purpose of this study was to determine if an acute 8 g dose of CM or 1.5 g dose of Nitrosigine increased brachial artery diameter over baseline following FMD.

## Method

### Participants

Twenty-four total participants, 16 men and 8 women, were recruited to participate from a land grant institution in the Midwest. All procedures were approved by the Institution Review Board and all participants signed a written informed consent before any testing began. Participation in the study was open to healthy, regularly exercising young adults between the ages of 18 and 30 years. Exclusionary criteria were as follows: hypertension, metabolic disorders, consumption of any pre-workout supplements six months prior to study participation, nicotine consumption in any of the previous six months, use of any prescription medications that have the potential to influence vasodilatory response, or use of any prescription drugs that influence the menstrual cycle. Screening for metabolic disease, tobacco use, and prescription drug use was conducted using a standard medical questionnaire adapted to an online survey form. Participants’ physical activity levels were assessed using the International Physical Activity Questionnaire (IPAQ short form). All participants were considered to have high levels of physical activity; they reported vigorous exercise on more than three days per week and exceeded 1500 (MET x min/week) or reported physical activity of at least 3000 (MET x min/week). Anthropomorphic, blood pressure, and heart rate data are found in Table 1.

**Table 1:** Participant Characteristics

Variable	Total Sample	Female	Male	*p*-value
		*n* = 6	*n* = 15	Sex Differences
Age (years)	22.3 ± 1.2	22.0 ± 0.8	22.4 ± 1.4	.44
Weight (kg)	73.66 ± 15.99	61.82 ± 8.14	78.83 ± 19.95	.02
Height (cm)	175.25 ± 9.83	163.28 ± 3.51	180.48 ± 6.38	< .001
Systolic BP (mmHg)	121.41 ± 13.07	111.71 ± 11.41	125.93 ± 11.47	.013
Diastolic BP (mmHg)	73.82 ± 9.53	71.71 ± 9.12	74.80 ± 9.86	.49
Heart rate (bpm)	68.50 ± 14.05	62.60 ± 15.17	70.60 ± 14.56	.31

Note. *BP* blood pressure; *mmHG* millimeters of Mercury. An independent samples t-tests was used to determine differences between sexes

### Research design

This study used a double-blind randomized, placebo controlled, crossover research design. The order of supplementation was randomized to control for order effects. Additionally, a third-party researcher who was not involved in data collection or analysis mixed and labeled all supplement drinks; codes were revealed after all data collection and analyses were complete. Participants were asked to complete the experimental protocol in a 2-h fasted state, verified by self-reports, and were asked to abstain from high fat foods, caffeine, and all other dietary or vitamin supplements 24 h prior to testing. Participants recorded their dietary habits for 24 h leading up the first trial, and diet summaries were sent to participants 24 h prior to subsequent trials to aid them in replicating their diet as accurately as possible before the second and third trials. The short dietary questionnaire (SDQ) was administered to estimate macronutrient consumption. SDQ assessed the servings of 36 most commonly occurring food consumed in the previous 24 h. Each participant was presented with the 36-item list and were asked to indicate the number of servings they had consumed within the previous 24-h period. Macronutrient and total caloric values were calculated based on publicly available nutrient profiles published by the USDA [22]. Each participant’s brachial artery FMD was assessed twice during each trial, once before supplement ingestion, and again 60 min post-ingestion. Male participants were required to wait for a minimum of seven days between trials as a supplement washout period and were tested around the same time of day (i.e. early morning, morning, mid-day, early afternoon, etc.). Female participants reported for supplement trials outside of their follicular phase of the menstrual cycle so that results were not confounded by changes in serum estradiol, which can significantly attenuate flow-mediated dilation response [23]. Because female participants allowed for approximately 28 days between trials, no further washout period precautions were taken. Otherwise, the procedure for female participants did not differ from the male participants. Sixty minutes after supplement consumption, each participant was asked which supplement they thought they had consumed. Participants were also asked whether they experienced any adverse side effects.

### Supplementation

All trials were completed with a minimum 7-day washout period before the following supplementation. Female participants were scheduled outside of their follicular phase, minimum of 48 h after termination of menses to avoid confounding hormonal effects [24]. The average time between trials was 24 days. The supplement doses administered were 8 g of CM (Bulk Supplements, Henderson, Nevada), 1.5 g of Nitrosigine (inositol-stabilized arginine silicate; Nutrition 21, Purchase, New York), and 8 g of dextrose, all of which were purchased in powder form and mixed with 16 oz of fruit punch flavored water in red-tinted shaker bottles to mask the differences in taste and color between the supplements. Eight grams of dextrose were also added to the CM and Nitrosigine supplement drinks to keep the caloric load similar between trials. Following baseline FMD and subsequent supplementation, the participants remained seated for 60 min to allow the supplement enough time to pass through the digestive tract, at which point plasma arginine levels should have been elevated [9].

### Brachial artery flow mediated dilation

Upon reporting for trials, participants assumed a supine position in a reclining phlebotomy chair with his or her left arm abducted approximately 70 degrees and supinated on an adjustable arm rest. Blood pressure and heart rate were recorded from the participant’s right arm every 5 min until hemodynamic values were stable. The experimenter then measured, marked, and recorded cuff and transducer landmarks on the participant’s left arm with a surgical marker to ensure that the ultrasound transducer and occlusion cuff were placed in the same location for each trial. The rapid inflation occlusion cuff (E20 and AG101 rapid cuff inflator, Hokanson, Bellevue, WA) was placed 2 cm distal from the antecubital fold, and a 10 MHz ultrasound transducer (LOGIQ e, GE Healthcare, Chicago, IL, USA) was placed at least 5 cm proximal but no more than 10 cm proximal from the antecubital fold. Dual mode Doppler ultrasound was used to simultaneously record blood flow velocity, which would later be used in calculations of maximum shear rate and shear rate area under the curve to maximum (AUC). After maintaining a stable image of the brachial artery, in which both arterial walls were clearly visible, two minutes of video elapsed providing a sufficient amount of time for the calculation of an average baseline diameter. Following the 2-min baseline period, the occlusion cuff was rapidly inflated to 250 mmHG and maintained for five minutes. After the 5-min occlusion phase, the cuff was rapidly deflated and changes in artery diameter and blood flow velocity were monitored for a 3-min period. Identical procedures were used for the baseline and post-supplementation FMD measurements. Generally, FMD procedures were determined using recommendations from Harris et al. [25]. All FMD measurements and AUC calculations were completed using Quipu Cardiac Suite software (Quipu, Pisa, Italy). Video was recorded live from the ultrasound monitor at 60fps, 1040 x 720p using screen capture software during the entire 10-min FMD procedure. Our intra-rater reliability was excellent (*r* = .93), similar to previously published data (ICC 0.84–0.99) [26].

### Analysis of flow-mediated dilation

An experimenter blinded to the supplement ID codes and the trial numbers of the videos individually analyzed each FMD video recordings using Quipu FMD Studio software (QUIPU, Pisa, Italy). By identifying the walls of the artery and calculating the average brachial artery diameter measurements in real time, the software was able to provide an accurate measure of arterial diameter for every second of video (20 measurements per second of video). Baseline diameters (D_Base_) reported in this study are averages of the brachial artery diameter during the entire 2-min pre-occlusion phase, whereas maximum diameter (D_peak_) is the largest recorded diameter within the 3-min period following the immediate release of the occlusion cuff. The FMD percentage (FMD%) was calculated using the following equation: (D_peak_ (mm) / D_Base_ (mm)) × 100. Shear rate was determined from the equation: (blood flow velocity (cm / sec) / diameter (cm)) for which the values are expressed in units of sec^− 1^, and shear rate AUC to maximum was calculated by taking the first integral of the shear rate curve from cuff release to D_peak_.

### Statistical analyses

All statistical analyses were conducted using Statistical Package for the Social Sciences (SPSS; IBM Corp, v.25; Armonk, NY). To determine potential sex differences in the response to supplementation, a 2 (sex) × 2 (time) × 3 (supplement) repeated measures analysis of variance (ANOVA) was used. There was no significant interaction with participant sex, so data were compiled and analyzed as one sample (*n* = 21) for the remainder of the statistical analyses. A 2 (time) × 3 (supplement) repeated measures ANOVA was used to test for significant differences in FMD%, D_base_, D_peak_, maximum shear rate, and shear rate AUC to maximum. Nutrition data from the SDQ were analyzed according to Shatenstein and colleagues [22]. A repeated measure ANOVA was used to analyze mean differences in total calorie (kcal), protein (g), carbohydrate (g), and total fat (g) intake variables between trials.

Examinations of the relationship between FMD baseline diameter (D_base_) and FMD% have found that there is a significant negative relationship between the two variables, which can unintentionally bias the interpretation of the study [27]. In the present investigation, an examination of the relationship between D_base_ and FMD% yielded a much weaker negative relationship; however, corrective analyses were conducted for comparison purposes. In line with the statistical analysis procedure used by Atkinson [27], the natural logarithm was calculated for D_base_ and D_peak,_ and the aggregated values were compiled into a predictive regression to determine the exponent of change. Corrected FMD % was obtained using the equation ((LnD_peak_/LnD_base_^0.94^)-1) × 100 for each FMD procedure.

## Results

**Fig. 1:**
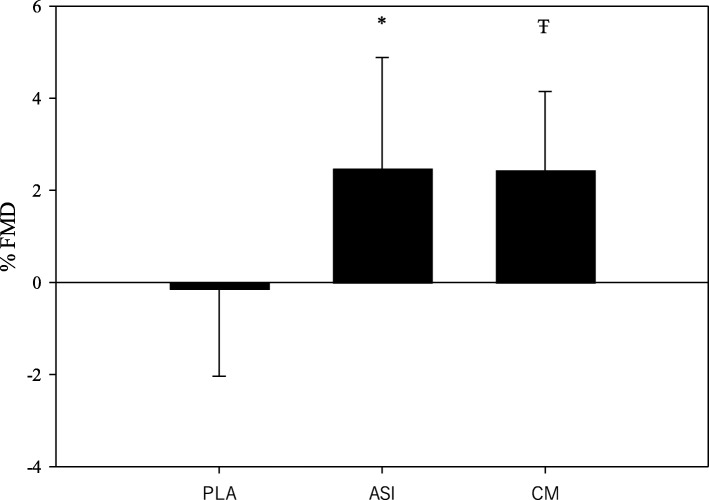
FMD percentages for each supplement group. Note. Results are presented as mean change post-supplementation; error bars are SD. * = Placebo group is significantly different from the Nitrosigine® group. Ŧ = Placebo group is significantly different than the citrulline malate group

**Fig. 2:**
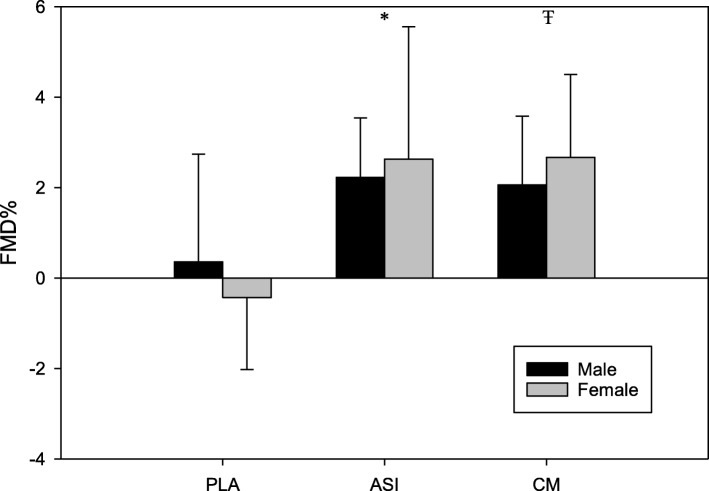
Scaled FMD percentages for each supplement group. Note. Results are presented as mean change post-supplementation; error bars are SD. * = Placebo group is significantly different from the Nitrosigine® group. Ŧ = Placebo group is significantly different than the citrulline malate group

**Fig. 3:**
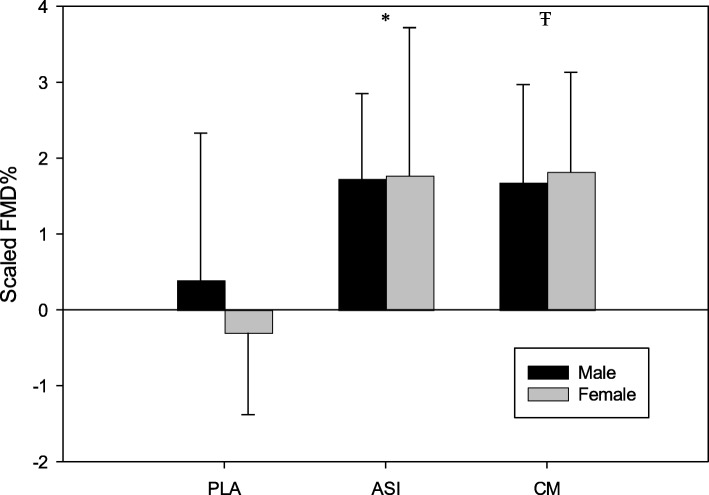
Correlation between baseline diameter and FMD%. Note. Results are presented as mean change post-supplementation; error bars are SD. * = Placebo group is significantly different from the Nitrosigine® group. Ŧ = Placebo group is significantly different than the citrulline malate group

A total of 21 young adults (*n* = 15 men and *n* = 6 women) completed all three supplement trials. A summary of the means, standard deviations, and ANOVA results for the FMD and estimated macronutrient intake data are located in Tables 2 and 3. For the unscaled FMD% values, there was a significant supplement x time effect (*F* = 11.64, *p* < .001; Fig. 1). Post-hoc analysis showed that Nitrosigine and CM supplementation resulted in significantly greater increases in FMD% when compared to the placebo trial; however, there was no significant difference between the two supplements or between sexes (Fig. 2). These findings were unchanged after allometric scaling (*F* = 10.61, *p* < .001; Fig. 3). CM increased FMD% from baseline by an average of 2.44% (95% CI: 1.66–3.2; Fig. 2) and Nitrosigine increased FMD% from baseline by an average 2.48% (95% CI: 1.38–3.57; Fig. 2). Statistical analysis found no significant supplement x time interaction for baseline diameter, maximum shear rate, or shear rate AUC to maximum. There were no significant differences in total calorie (kcal), protein (g), or total fat (g) consumption in the 24-h period before each trial; however, total carbohydrate consumption was higher in the 24 h prior to the Nitrosigine trial than what preceded the CM and placebo trials (*F* = 3.327, *p* = .047).

**Table 2:** ANOVA results for all FMD variables

Variable	Pre-Supplement	Post-Supplement	*p*-value Supplement x Time Interaction	*p*-value Supplement Effect
Flow-Mediated Dilation (%)				
Citrulline Malate	7.15 ± 2.44	9.60 ± 2.72	< .001	< .001^a^
Nitrosigine	8.00 ± 3.14	10.48 ± 2.63		< .001^a^
Placebo	8.01 ± 2.90	7.87 ± 2.83		.76^b^
Allometric Scaled FMD %				
Citrulline Malate	7.09 ± 1.80	8.85 ± 1.98	< .001	< .001^a^
Nitrosigine	7.65 ± 2.16	9.47 ± 1.84		< .001^a^
Placebo	7.69 ± 2.17	7.65 ± 2.07		.90^b^
AUC Hyperemic Shear Rate to Maximum (∫ sec^−1^)				
Citrulline Malate	164,109 ± 175,726	269,706 ± 217,793	.30	.01^a^
Nitrosigine	245,539 ± 190,582	288,877 ± 199,491		.29^b^
Placebo	318,179 ± 205,705	311,804 ± 224,332		.92^b^
Maximum Shear Rate (sec^−1^)				
Citrulline Malate	771.97 ± 310.45	786.70 ± 253.42	.86	.75^a^
Nitrosigine	812.08 ± 376.58	790.26 ± 268.38		.76^a^
Placebo	918.25 ± 490.64	914.91 ± 367.76		.99^a^

*Note. FMD* flow-mediated dilation. Values are reported as mean + *SD*. Values with different letters represent statistically significant values

**Table 3:** Nutrient Consumption between Supplement Trials

	Supplement		ANOVA (Nutrient (1) x Supplement (3))
Total Energy (kcal)	Citrulline Malate	2615.30 ± 1105.40	*F* = 2.00, *p* = .15
	Nitrosigine®	3103.00 ± 1310.64	
	Placebo	2921.27 ± 1402.11	
Protein (g)	Citrulline Malate	133.85 ± 47.78	*F* = 0.34, *p* = .71
	Nitrosigine®	139.82 ± 49.93	
	Placebo	142.94 ± 54.97	
Carbohydrate (g)	Citrulline Malate	283.84 ± 163.51	*F* = 3.33, *p* = .047
	Nitrosigine®	376.47 ± 196.54	
	Placebo	328.22 ± 184.43	
Total Fat (g)	Citrulline Malate	107.49 ± 47.12	*F* = 0.98, *p* = .39
	Nitrosigine®	120.05 ± 51.98	
	Placebo	118.48 ± 62.34	

*Note. kcal* kilocalorie; *g* grams. Values are reported as mean + *SD*. *P*-values represent differences between trials

## Discussion

The purpose of this study was to determine whether acute Nitrosigine or CM supplementation effected the NO producing capacity of endothelium dependent vasodilation as measured by change in FMD. Limited studies exist examining the effect of CM on endothelium dependent vasodilation [15] and results have been inconclusive compared to the observed ergogenic effects on high intensity exercise [11, 13, 14, 16]. Meanwhile, the present study, to our knowledge, is the first to examine the acute effects of Nitrosigine supplementation on endothelium dependent vasodilation as measured by FMD. Results from this study indicate that both 1.5 g Nitrosigine and 8 g CM significantly increase vasodilation in response to FMD. Although CM and Nitrosigine increase serum arginine through slightly different mechanisms, both appear to be equally effective at increasing endothelium response to shear stress. By scaling the data to control for the bias introduced by baseline diameter variation, we can safely conclude that the observed changes were not unduly influenced by statistical flaws.

Research has firmly established that Nitrosigine and CM are both effective at increasing serum arginine levels following both acute and continuous supplementation [9, 18, 28, 29]. However, previous research that sought to increase serum arginine and subsequently test for changes in FMD, have failed to find significant changes after supplementation [5, 18, 30, 31]. Potential differences in baseline serum arginine may explain a portion of the variability in FMD results among L-arginine and L-citrulline studies. When baseline arginine and FMD% are low, whether due to a lack of dietary protein or cardiovascular pathology, supplementing to increase serum arginine drastically improves FMD response and other cardiovascular markers [32, 33]. In the present investigation, participants had average protein intake values of 0.94 g/kg/day, which is above the recommended dietary allowance (RDA) of 0.80 g/kg/day. Additionally, participants with increased cardiovascular disease risk and reduced levels of cardiovascular fitness respond differently to increases in serum arginine, and thus experience a dampened FMD response [34]. Although baseline serum arginine levels were not assessed in the present investigation, it is expected in the current sample of young and physically active adults that normal baseline arginine levels existed [35]. Given the large number of variables that have the potential to influence endothelial function, sample homogeneity is an essential factor in determining the presence of a beneficial vasodilatory effect, especially given small sample sizes in many supplement studies [36]. FMD is enhanced among individuals that have higher levels of cardiorespiratory fitness and higher levels of physical activity [37–39]. The sample recruited for the present investigation was similar in age, and all reported high levels of physical activity (3847 MET-min/week) Consequently, the supplement effects observed in this study may be limited to highly trained young adults. Further investigation, specifically a comparison study of younger and older athletes and non-athletes will be necessary to verify this possibility.

## Conclusions

Results from this study support a novel finding that acute supplementation with CM and Nitrosigine can improve endothelial-dependent vasodilation in trained young adults. Prior to this study, there was no comparison of CM and Nitrosigine effects using FMD. This study supports previous research findings that Nitrosigine may be a beneficial pre-workout supplement, and that a 1.5 g dose of Nitrosigine may be equally as effective at increasing endothelial response as a larger 8 g dose of CM.

## Data Availability

The data generated and analyzed during this study are available from the corresponding author upon request.
